# Structural studies of the spliceosome: Bridging the gaps

**DOI:** 10.1016/j.sbi.2022.102461

**Published:** 2022-12

**Authors:** J. Tholen, W.P. Galej

**Affiliations:** European Molecular Biology Laboratory, 71 Avenue des Martyrs, 38042 Grenoble, France

## Abstract

The spliceosome is a multi-megadalton RNA–protein complex responsible for the removal of non-coding introns from pre-mRNAs. Due to its complexity and dynamic nature, it has proven to be a very challenging target for structural studies. Developments in single particle cryo-EM have overcome these previous limitations and paved the way towards a structural characterisation of the splicing machinery. Despite tremendous progress, many aspects of spliceosome structure and function remain elusive. In particular, the events leading to the definition of exon–intron boundaries, alternative and non-canonical splicing events, and cross-talk with other cellular machineries. Efforts are being made to address these knowledge gaps and further our mechanistic understanding of the spliceosome. Here, we summarise recent progress in the structural and functional analysis of the spliceosome.

## Introduction

**Figure 1:**
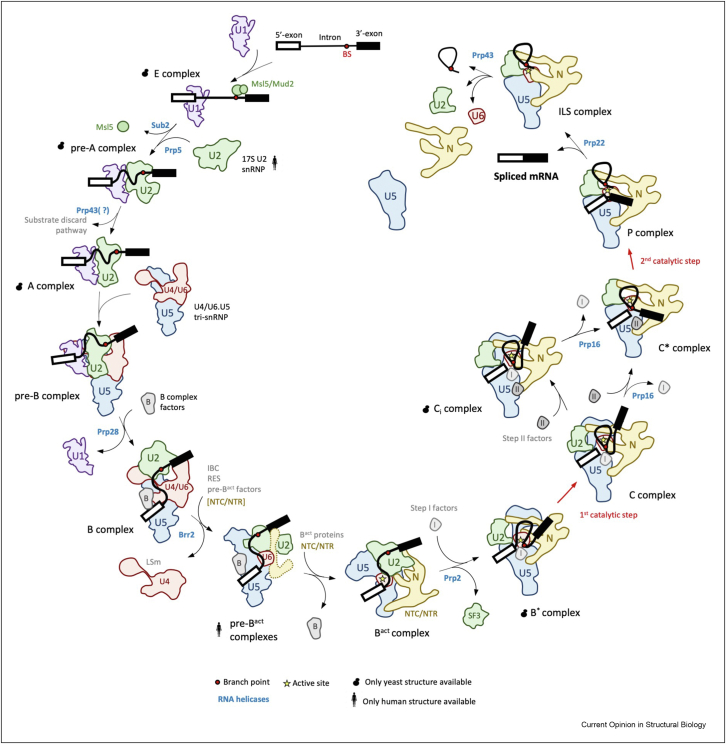
Stepwise assembly of yeast (*Saccharomyces cerevisiae*) and human spliceosomes from snRNPs and *trans*-acting factors. For simplicity, only factors relevant to this review are indicated. Cartoon shapes of splicing complexes are based on yeast structures, except the 17S U2 snRNP and pre-B^act^ complex, for which only human structures are available. Please note, that there are some differences between yeast and human splicing pathways which were not depicted here.

In eukaryotes, most genes are transcribed as precursors of messenger RNAs (pre-mRNAs), wherein protein-coding segments, exons, are interrupted by non-coding regions, introns. Intron removal is catalysed by the spliceosome, a large macromolecular complex consisting of several dozen proteins and five snRNAs, largely pre-associated as the small nuclear ribonucleoprotein particles (snRNPs) [1]. The spliceosome assembles *de novo* on each intron in a stepwise manner (Figure 1). During this process, U1 snRNP and U2 snRNPs recognise the 5′-splice site (5′-SS) and the branch site (BS), effectively defining exon-intron boundaries and allowing the recruitment of the U4/U6.U5 tri-snRNP to form a pre-catalytic pre-B complex. The pre-B complex undergoes a series of ATP-dependent rearrangements leading to the formation of the RNA catalytic core and juxtaposition of the splice sites at the active site, which allows a two-step transesterification reaction to join the coding sequences (exons) together into a mature mRNA [1].

The dynamics and complexity of the splicing machinery posed a great challenge for structural studies, in particular by X-ray crystallography, which required extensive engineering of RNAs, proteins and crystal contacts [2]. Developments in electron cryo-microscopy (cryo-EM) have overcome these limitations and paved the way towards the first high-resolution structures of fully assembled spliceosomes [3, 4, 5, 6]. Since then, many of the key spliceosome assembly intermediates have been characterised structurally, providing unprecedented insights into the mechanism of pre-mRNA splicing [7,8]. Recent efforts have been focussing on dissecting conformational transitions into finer steps, investigating coupling between the transcription and splicing machinery as well as the non-canonical, U12-dependent splicing pathway. There have also been substantial improvements in the resolution limits of spliceosome reconstructions, providing ever more accurate structural description of splicing complexes. Here, we summarise recent progress in the mechanistic understanding of the pre-mRNA splicing and outline future directions.

## Dissecting early splicing events

Recognition of exon-intron boundaries is achieved largely during the early stages of spliceosome assembly and has a profound impact on the fidelity of the splicing reaction. The 5′-SS is recognised by the U1 snRNP, while the BS and polypyrimidine tract (PPT) are recognised by SF1 (Msl5 in yeast) and U2AF2 (Mud2 in yeast), respectively. The resulting early (E) complex is the first ATP-independent spliceosome assembly intermediate [9,10]. In mammals, complex E contains loosely associated U2 snRNP [11] and requires ATP-dependent remodelling to form base-pairing interactions between U2 snRNA and the BS, which results in the A complex. The cryo-EM reconstruction of the *Saccharomyces cerevisiae* A complex stalled by a BS mutation that prevents its further assembly, provided first insight into its bi-lobal architecture and contacts between U1 and U2 snRNP components bridging the two splice sites together [12]. Several of the yeast U1 snRNP components, although conserved in humans, are not stably associated with the human U1 snRNP and act independently as alternative splicing regulators [12,13]. These include Nam8 (human TIA-1), Luc7 (human LUC7L), Snu71 (human RBM25) as well as Prp39 (human PRPF39) and Prp40 (PRPF40A), which play a role in bridging 5′SS-BS interaction [12,14]. The structure of complex A in higher eukaryotes remains unknown. This is of particular interest in humans due to extensive regulatory mechanisms involved in the splice site selection (i.e. alternative splicing) and diseases associated with aberrantly spliced gene products [15,16].

**Figure 2:**
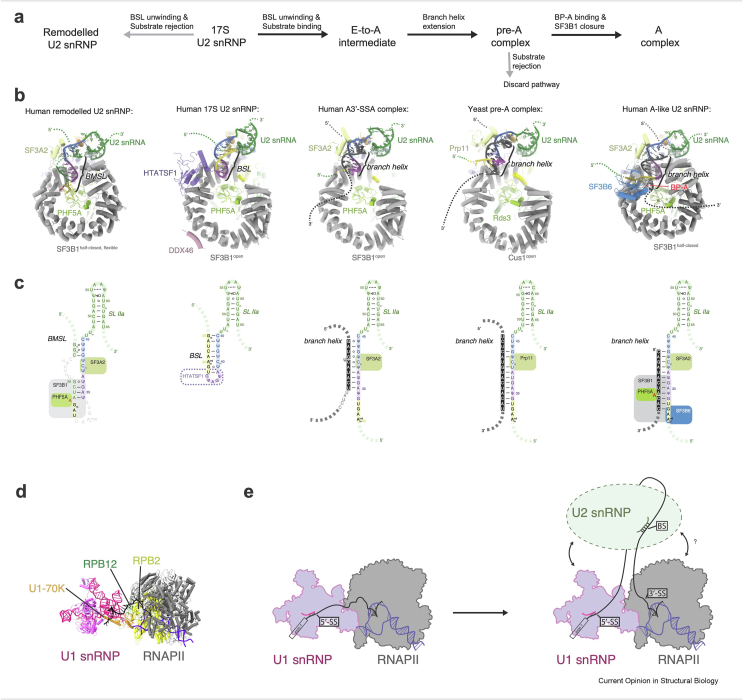
Structural insights into early splicing events. **(a)** Proposed order of events during branch site recognition. **(b)** U2 snRNP 5′-domain structures from recently reported early splicing complexes with highlighted key protein factors and RNA elements [17,18,25,31]. **(c)** Schematics of RNA secondary structures from the corresponding complexes showing the key transitions and interactions between U2 snRNA and the pre-mRNA substrate. **(d)** The structure of the U1 snRNP-RNAPII complex determined by cryo-EM, showing the stable interfaces between components of the two complexes [42]. **(e)** Proposed intron looping model for co-transcriptional splicing and formation of the spliceosomal A complex.

Several recent studies have addressed the mechanism of early splicing events using biochemical and structural approaches. The structure of the human 17S U2 snRNP before substrate binding confirmed that the U2 snRNA forms a branch point-interacting stem loop (BSL) [17,18], which was previously inferred from biochemical and genetic studies [19] (Figure 2). Integrity of the BSL is necessary for it to act as the branch sequence receptor within 17S U2 snRNP [19]. HTATSF1 (yeast Cus2) binds a linear U2AF ligand motif (ULM) in SF3B1 (yeast Hsh155) via its U2AF homology motif (UHM) domain [20]. Similar UHM-ULM interactions are utilised by several different splicing factors, in particular during early splicing events [21]. In the 17S U2 snRNP, the HTATSF1 RRM domain forms a stable interface with an open state of SF3B1 and appears to play a critical role in maintaining the global conformation of the U2 snRNP by occupying the branch helix interaction site on the SF3B1 HEAT repeat domain (SF3B1^HEAT^) and stabilising the BSL [22,23].

The structure of the human complex E remains unknown, most likely due to its intrinsic dynamic nature and the heterogeneity associated with the regulation of early splicing events. Nevertheless, it is anticipated that the U1 and U2 moieties of complex E would remain similar to the U1 snRNP:5′-SS complex and 17S U2 snRNP structures determined in isolation [17,18,24]. What is less clear is how U1 snRNP and branch point-binding proteins (SF1, U2AF2) are bridged together and what is the mechanism of the BS handover from SF1/U2AF2 to the U2 snRNP and subsequent branch helix formation. Some insight into the latter was gained by inhibiting branch helix formation in human nuclear extract with spliceostatin A (SSA) to form the A3′-SSA complex, a variant of the pre-A complex [25]. The high-resolution structure of the U2 snRNP moiety of the A3′-SSA [25] suggests that the branch helix may be formed by a toehold-mediated strand invasion mechanism, wherein a short stretch of intron sequence complementary to the BSL is gradually extended towards the U2 3′-end via strand invasion. Eventually, this leads to the formation of a fully-fledged branch helix [25] (Figure 2). Given the very low conservation of branch site sequences in humans [8], it is unclear whether this mechanism applies to all BS sequences.

BS recognition by the U2 snRNP was recently reconstituted *in vitro,* allowing isolation of the U2 snRNP moiety of the human A complex, the A-like U2 snRNP [18]. The structure of this complex revealed atomic details of how, after HTATSF1 is displaced, the fully formed branch helix inserts the bulged-out BP-A into the binding pocket formed by SF3B1^HEAT^ and PHF5A, causing a transition of the HEAT repeats into a half-closed conformation. In this state, SF3B6 binds U2 snRNA at the end of branch helix providing additional stabilization that may be needed by BS sequences that do not have strong U2 snRNA base pairing potential [18] (Figure 2). Noteworthy, both A3′-SSA and A-like U2 snRNP complexes were assembled on a BS with good complementarity to the U2 snRNA, therefore it remains to be seen how other, weakly complementary BS would be accommodated within the SF3b cavity. It was previously shown that the interactions between the branch helix and its SF3B1 binding pocket play important roles in BS selection and numerous disease-associated mutations have been identified in SF3B1 [26, 27, 28, 29].

Incubation of the human 17S U2 snRNP with ATP in the absence of a substrate has revealed a new conformational state, in which the U2 snRNA forms a branch helix-mimicking stem loop (BMSL) that interacts with the SF3b complex in a manner similar to the branch helix [18]. ATP-dependent competition between productive and non-productive conformational changes during branch site selection in yeast was previously postulated to act as a BS fidelity control checkpoint [30]. The BMSL may come into existence when a substrate is rejected due to the low stability of the resulting branch helix, or it may be a ground state of the U2 snRNP, before it is activated to form the BSL and becomes capable of BS binding (Figure 2).

Another BS fidelity checkpoint was revealed by biochemical and structural studies of the yeast pre-A complex that was assembled on a substrate missing the bulged-out branch point adenosine (BP-A) [31,32]. In this configuration, the branch helix is fully formed, but does not bind SF3B1^HEAT^, which remains in the open conformation [31]. Simultaneously, Prp5 (human DDX46) blocks recruitment of the U4/U6.U5 tri-snRNP, consistent with previous biochemical data [33]. Transition from the open to the closed conformation of SF3B1 would likely induce displacement of Prp5 from the pre-A complex and allow subsequent steps of spliceosome assembly [31].

Recent biochemical data shows that DHX15 (yeast Prp43) mediates the disassembly of the early spliceosomes containing U2 snRNP [34] and could in principle target non-functional pre-A complexes [31]. G-patch proteins, such as SUGP1, which was shown to modulate BS and 3′-SS usage, could play a role in this process by regulating DHX15 activity [27,35].

## Cross-talk with the transcription machinery

Transcription and splicing are highly dependent on one another and functional coupling between the two processes is well documented [36,37]. Consequently, most introns are spliced immediately or soon after they emerge from the elongating RNAPII [38, 39, 40], although the exact timing of these events is transcript-dependent and remains a subject of discussion [41]. In contrast to the functional data on the temporal aspect of co-transcriptional splicing, much less is known about the physical interactions between the two machineries.

A recent report by Zhang et al. describes the structure of the U1 snRNP assembled *in vitro* with the RNAPII and provides first insights into the structural basis of coupling between transcription and splicing complexes (Figure 2d) [42]. The structure revealed that U1-70K forms a stable interface with two RNAPII subunits, RPB2 and RPB12, positioning the 5′-SS close to the RNA exit channel of RNAPII. This supports a previously proposed model that 5′-exon might be kept in this position while the nascent RNA is looped out by the elongating polymerase (Figure 2e) [43]. An extension of this model would suggests that RNAPII may also facilitate recruitment of the U2 snRNP, which could then bind the branch site as soon as it emerges from the exit channel. It is not clear if such a complex exists in cells, but tight spatial coupling is consistent with the very short splicing times observed for many mammalian and yeast introns; for these, splicing reactions can occur even before the downstream exon is fully transcribed [38, 39, 40]. More studies are required to validate the functional significance of the observed RNAPII-U1snRNP interface for pre-mRNA processing events.

Notably, it was previously proposed that splice sites in long mammalian introns could be initially defined across short exons (exon-definition model) before being converted into intron-defined complexes [14,44]. It remains to be seen if both models can be reconciled into one unified mechanism.

## Known pathway, new states

### Spliceosome (pre)activation

**Figure 3:**
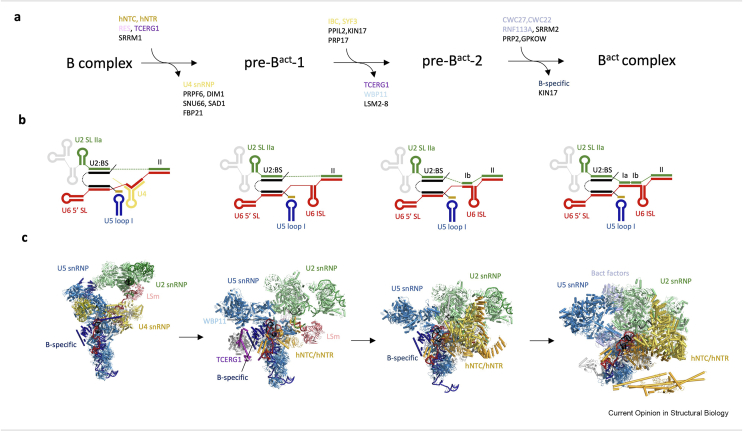
Spliceosome activation and the pre-Bact complexes. **(a)** Schematics depicting the key proteins and RNAs recruited to or displaced from the spliceosome during activation, based on the structures of human B [46,47], pre-B^act^-1 and pre-B^act^-2 [45] and B^act^ [50,51] complexes. **(b)** Schematic representation of RNA secondary structure in the corresponding complexes highlighting progressive formation of the U2/U6 catalytic core. **(c)** Molecular models of the B, pre-B^act^-1, pre-B^act^-2 and Bact complexes showing large scale global conformational changes in the corresponding complexes.

Formation of the spliceosome active site (termed activation; the B-to-B^act^ transition) involves displacement of ∼25 proteins and U4 snRNA followed by recruitment of more than two dozen new factors [1]. This allows the formation of the U2/U6 catalytic core of the spliceosome, which binds the catalytic metal ions. Given the magnitude of this conformational change, it would be unlikely to happen without any intermediate states. In their recent report, by using small molecule splicing inhibitor, Townsend et al. captured two novel intermediate states of the spliceosome (pre-B^act^-1 and pre-B^act^-2) during B-to-B^act^ transition [45]. Although the mechanism of this inhibition could not be explained, it allowed the authors to dissect spliceosome activation into finer steps to better track the trajectories of individual proteins and RNAs (Figure 3a). Large-scale conformational movements of the U2 snRNP and the Brr2 helicase were captured in an intermediate position and several splicing factors were visualised for the first time (i.e. TCERG1, KIN17, WBP11), giving insight into their possible functions [45] (Figure 3a and c).

The pre-B^act^-1 and pre-B^act^-2 complexes were captured after the release of U4 snRNA, but they maintained B complex-specific proteins [46,47]. As a consequence, Prp8, the key scaffolding protein of the spliceosome, remains in the open conformation [48,49] allowing progressive formation of the U2/U6 RNA catalytic core in the active site cavity [45]. In pre-B^act^-1, the internal stem-loop of the U6 snRNA (ISL) is formed, but the rest of the catalytic core remains disordered (Figure 3b). This partially formed core is stabilised by the B complex-specific protein WBP11, which presumably acts as a chaperone and needs to be displaced to allow U2/U6 helix Ib formation in complex pre-B^act^-2. Finally, the transition to B^act^ allows stable docking of the U2/U6 helix Ia onto the surface of Prp8 and Prp8 domain closure [50,51]. It should be noted that the limited resolution of these reconstructions leaves some ambiguity as to the exact snRNA base-paring patterns described for these new intermediates. Interestingly, nearly all proteins needed to stabilise the catalytic core in B^act^ complex are already pre-recruited to the open conformation of Prp8 in both pre-B^act^ complexes, hence new structures provide insights into the order of protein assembly and causative links of these conformational transitions.

To complete the activation process, the B^act^ complex needs to be further remodelled to B^∗^, in which the 5′-SS and BS are juxtaposed. New structural data support the role of the G-patch protein Spp2 in recruiting DEAH-box helicase Prp2 to the B^act^ spliceosome to mediate this final transition [52,53]. However, the exact mechanism by which Prp2 achieves this goal is still not well understood.

### C_i_ intermediate

The single active site of the spliceosome is responsible for catalysing both steps of splicing. Consequently, one of the first step products, the intron lariat branch site, needs to be replaced from the active site by the 3′-exon for the second catalytic step. The remodelling leading to this exchange is catalysed by the DEAH-box ATPase Prp16 [54], which pulls the intron and allows for the exchange of proteins specific to branching (i.e. Yju2, Cwc25, and Isy1) with those specific for exon ligation (Prp18, Slu7, and Prp17) [55,56].

It has been proposed that these two sets of factors modulate the equilibrium between catalytic steps of splicing [57]. Exon ligation factors can bind the spliceosome already at the B^∗^ stage, however, the exact mechanism of this recruitment remained unclear [58]. In the recent report, Wilkinson et al., performed a thorough analysis of multiple cryo-EM datasets of yeast catalytic spliceosomes and identified a previously unknown intermediate—the C_i_ complex [59]. The C_i_ complex has a conformation nearly identical to the C complex [5], but contains pre-recruited exon ligation factors, Prp18 and Slu7 (Figure 1). This demonstrates that branching and exon–ligation factors can be bound in one complex, consistent with biochemical studies [58]. Pre-recruited exon ligation factors prime the C complex for the remodeling and ensure immediate stabilization of the C^∗^ conformation, preventing reversal of this change. Therefore, they influence conformational equilibrium between the two catalytic steps of splicing.

## The minor splicing pathway

A small subset of introns contain non-canonical splice sites, which require a distinct machinery for accurate processing [60,61]. These so-called U12-dependent introns are relatively rare, but are located in genes with critical cellular functions and are widespread in eukaryotes [62,63]. Removal of the U12-dependent introns is conducted by the minor spliceosome, which contains four unique snRNAs (U11, U12, U4atac, and U6atac) that substitute for their major spliceosome counterparts (U1, U2, U4, and U6). The U5 snRNP and numerous proteins are likely shared between the two systems, however, proteomic data for the minor splicing complexes is largely missing due to their scarcity in cells [64, 65, 66].

**Figure 4:**
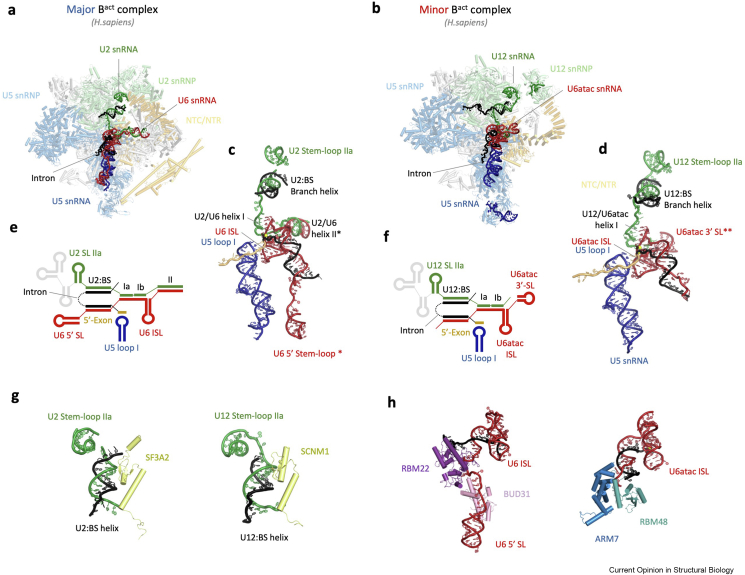
Structure of the minor spliceosome. **(a)** Overall architecture of the major spliceosome B^act^ complex with RNA elements highlighted in the foreground [67]. **(b)** Human minor spliceosome B^act^ complex [50]. **(c)** and **(d)** Structures of the RNAs in the major and minor B^act^ complexes showing remarkable structural similarity despite divergent sequences. **(e)** and **(f)** Schematics of the RNA secondary structure elements present in the major and minor B^act^ complexes. Elements exclusive to the major or minor spliceosome are indicated with ∗ and ∗∗, respectively. **(g)** and **(h)** Distinct proteins likely serve similar functions in the major and minor splicing systems.

The structure of the minor spliceosome B^act^ complex was recently reported [67] showing overall good agreement with yeast and human major B^act^ complexes (Figure 4a and b). In particular, the organisation of RNA elements at the active site and metal ion coordination is well conserved (Figure 4c and d) with the exception of the U2/U6 helix II and U6 5′ stem-loop (5′-SL), which are both missing in the minor splicing system. The former is likely substituted by the U6atac 3′ stem-loop (3′-SL), located in a similar position (Figure 4c–f). Importantly, the structure of the minor B^act^ complex revealed several novel components, which are unique to the minor splicing pathway and shed light on the functional mimicry between components of both spliceosomes.

One of the novel components, SCNM1, wraps around the entire U12 snRNP and binds the branch helix in a manner resembling SF3A2 (yeast Prp11) and its C-terminal tail is placed where SF3A1 (yeast Prp21) is located in the major B^act^ complex. This suggests that SCNM1 functionally substitutes for the SF3a complex (Figure 4g). Two other factors, ARMC7 and RBM48, bind the 5′ end of the U6atac snRNA, recognise the characteristic γ-monomethyl phosphate cap structure, and play a role in positioning of the U6atac:5′-SS duplex and guiding the intron downstream from the 5′-SS. Therefore, these factors might fulfil a function similar to Prp17 and the NTR components RBM22 and BUD31 in the major B^act^ complex, which bind the 5′ stem-loop of U6 snRNA, an element that is missing in U6atac (Figure 4h).

The structure of the minor B^act^ complex provides a first glimpse into the architecture of a parallel splicing machinery and sets the stage for further investigations.

## Conclusions and future perspectives

In the past few years, structures of many stable assembly intermediates of yeast and human spliceosomes have been determined by cryo-EM [7,8]. Collectively, they provide unprecedented mechanistic insights into the inner workings of the spliceosome and establish a structural framework for future functional studies. However, due to their intrinsic dynamics, the insufficient resolution of many available reconstructions hinders accurate atomic modelling. Furthermore, large parts of the spliceosomes remain unresolved in cryo-EM reconstructions, even though their components are present in biochemical preparations. This could be due to intrinsic disorder or an artefact caused by sample preparation and vitrification procedures. In either case, there is room for substantial improvements in both areas. New approaches to the analysis of continuous movements/complex transitions in cryo-EM reconstructions could help in dealing with this problem and lead to more quantitative description of the underlying structural dynamics [50,68, 69, 70]. Accurate structure prediction with AlphaFold2 [71] will also allow for more unambiguous interpretation of the data at medium or low resolution and, in combination with other methods, yield more reliable structural models.

Further dissections of the transitions between different spliceosomes′ conformations will be crucial to understand the precise trajectories of all components of the system. More sophisticated biochemical approaches or time-resolved methodologies could play important roles in dealing with this issue. Structural insights into alternative splicing regulation as well as minor splicing pathways remain largely uncharted areas that need further investigations. Finally, most of the proteomic and structural data on spliceosomes is derived from a just a few model organisms and a limited number of pre-mRNA substrates. A wide variety of regulatory mechanisms specific to certain organisms and substrates remains to be investigated.

## Declaration of competing interest

The authors declare no conflict of interest.
